# Variation in Chloroplast Genome Size: Biological Phenomena and Technological Artifacts

**DOI:** 10.3390/plants12020254

**Published:** 2023-01-05

**Authors:** Ante Turudić, Zlatko Liber, Martina Grdiša, Jernej Jakše, Filip Varga, Zlatko Šatović

**Affiliations:** 1 Centre of Excellence for Biodiversity and Molecular Plant Breeding (CoE CroP-BioDiv), Svetošimunska c. 25, 10000 Zagreb, Croatia; 2 Faculty of Agriculture, University of Zagreb, Svetošimunska c. 25, 10000 Zagreb, Croatia; 3 Faculty of Science, University of Zagreb, Marulićev trg 9a, 10000 Zagreb, Croatia; 4 Biotechnical Faculty, University of Ljubljana, Jamnikarjeva 101, 1000 Ljubljana, Slovenia

**Keywords:** genome databases, chloroplast genome, sequence length, taxonomy

## Abstract

The development of bioinformatic solutions is guided by biological knowledge of the subject. In some cases, we use unambiguous biological models, while in others we rely on assumptions. A commonly used assumption for genomes is that related species have similar genome sequences. This is even more obvious in the case of chloroplast genomes due to their slow evolution. We investigated whether the lengths of complete chloroplast sequences are closely related to the taxonomic proximity of the species. The study was performed using all available *RefSeq* sequences from the asterid and rosid clades. In general, chloroplast length distributions are narrow at both the family and genus levels. In addition, clear biological explanations have already been reported for families and genera that exhibit particularly wide distributions. The main factors responsible for the length variations are parasitic life forms, IR loss, IR expansions and contractions, and polyphyly. However, the presence of outliers in the distribution at the genus level is a strong indication of possible inaccuracies in sequence assembly.

## 1. Introduction

Chloroplasts are cell organelles in which photosynthetic reactions occur. They have their own genome (cpDNA)—the plastome—which is generally described as circular [1], but in some cases has been described as a linear, multimeric circular or branched double-stranded molecule [2,3,4]. The plastome is usually 120 to 170 kbp long and consists of 120 to 130 genes (e.g., [5,6]).

The chloroplast genome sequence is a valuable source of data for evaluating plant evolution and taxonomy at different taxonomic levels [7]. Comparing the average chloroplast genome to the mitochondrial or nuclear genome, the gene composition is highly conserved with a collinear sequence arrangement. The slow evolution of most plastomes may be explained by the organization of chloroplast genes into operons, the mostly uniparental mode of inheritance, the activity of highly effective repair mechanisms, and very rare plastid recombination [8]. However, when there are major rearrangements in the chloroplast genome, they are usually associated with a parasitic and mycoheterotrophic lifestyle [9,10], and with an unusual mode of chloroplast inheritance, paternal or biparental [11]. Plant plastome size is specific to particular taxonomic groups such as order or family, but differences have very rarely been found between species of the same genus [12,13,14,15]. Three main factors are thought to be responsible for variation in chloroplast genome length: variation in intergenic regions (e.g., rice, family Pinaceae and genus *Oenothera*), variation in IR regions (e.g., gymnosperms, family Poaceae and Fabaceae), and gene loss (e.g., parasitic plants) [16].

However, there is no doubt that the cpDNA genomes of genetically close species are similar and that the conclusions derived should be valuable. There are numerous biological explanations for cases in which sequence similarity does not follow genetic proximity. Nevertheless, it should not be overlooked that some differences are due not to biology but to the technology used for sequence assembly. This is because the numerous different pipelines for genome sequence assembly do not always produce the same results. Therefore, observed differences between species of the same genus may indicate possible inaccuracies in the assembly process.

Advances in sequencing technologies have made genomic data more affordable. Together with the development of assembly methods, this has led to an increasing number of assembled genomes. This is clearly observed in the case of cpDNA data, as it is much easier to assemble chloroplast DNA than nuclear DNA. The reason for this is the higher copy number of cpDNA molecules [17,18] and the small size of the genome [1]. This is reflected in the number of genomes available in public databases. There is at least an order of magnitude more cpDNA than nuclear plant genomes in these databases [19]. At the time of writing, more than 10,000 plant cpDNA sequences were available in the NCBI (National Center for Biotechnology) Reference Sequence (*RefSeq*) collection, which provides a comprehensive, integrated, nonredundant, and well-annotated set of sequences. The number of cpDNA sequences in the NCBI *GenBank* database, which contains a collection of all publicly available DNA sequences, is a few times larger.

In general, bioinformatic solutions are developed based on biological knowledge and previous results on the subject; this is true for both chloroplast genome assemblers and annotation tools. Specialized chloroplast genome assemblers use existing results as reference sequences [20,21] or as starting positions for seed-and-extend assembly [22]. Therefore, assembly tools benefit from a larger pool of reference sequences from which to select the closest sequences, and the assembly process can be guided by their properties. Similarly, chloroplast annotation tools [23,24,25,26,27] rely on information from the gene database. In our opinion, the biological property of a slow evolutionary rate of the chloroplast genome combined with a large amount of available assembled sequence data may also be a fruitful combination to distinguish biological phenomena from technological artifacts.

We analyzed *RefSeq* chloroplast sequence length distribution using data from more than 5500 cpDNA sequences from plant species belonging to the asterid and rosid clades. The objectives of this study were to (a) assess the distribution of chloroplast sequence lengths at the family and genus levels, (b) identify families/genera with particularly wide distributions, and (c) detect outliers in the distribution. Possible explanations for wide distributions and the presence of outliers are provided. The utility of the results for the development of specialized chloroplast bioinformatics tools is discussed. We implemented a bioinformatics pipeline that can be used to analyze sequences of any taxa and store the result of each step in a concise format.

## 2. Results

### 2.1. Data Acquisition

**Figure 1:**
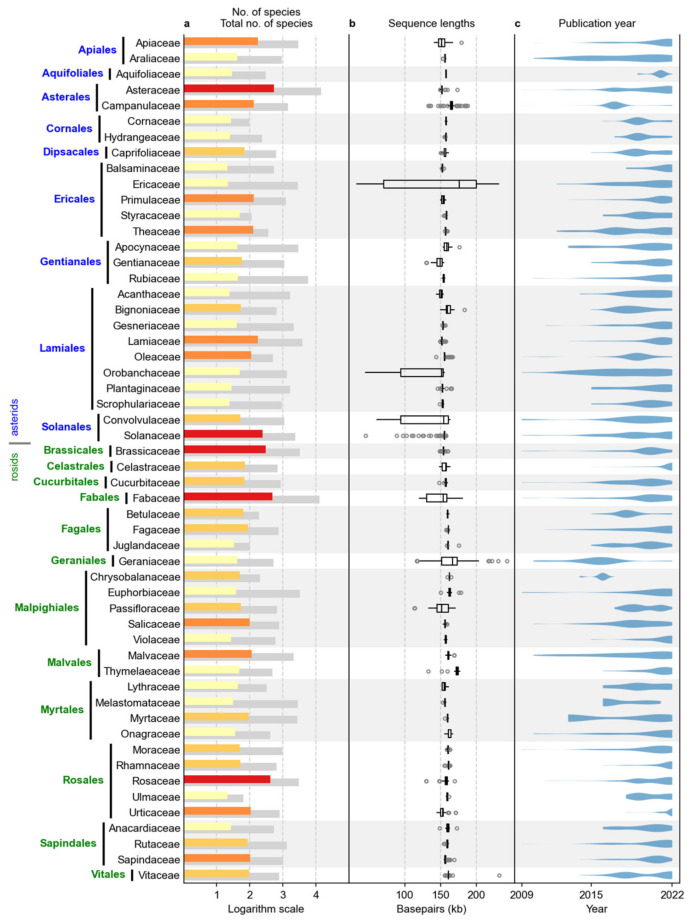
Number of *RefSeq* complete chloroplast sequences, distribution of sequence lengths, and publication years for the families of asterids and rosids containing 20 or more sequences. (**a**) Number of species according to NCBI taxonomy (gray bar) and number of available *RefSeq* chloroplast sequences (colored bar), where yellow represents 20–49, light orange 50–99, dark orange 100–199 and red ≥200 species. (**b**) Box plots of sequence lengths. (**c**) Violin plots of the number of published sequences by year.

A total of 5545 sequence summaries were acquired, of which 2534 and 3011 sequences were from the asterid and rosid clades, respectively (Table 1). The plant families with at least 20 sequences (the number chosen as the threshold) are shown in Figure 1. The data presented in the figure include 5076 sequences, representing 91.5% of the dataset, with asterid and rosid clades represented by 2337 and 2739 sequences, respectively. The largest families per clade were Asteraceae (asterids) and Fabaceae (rosids), with 543 and 480 sequences, respectively. In the NCBI Taxonomy Database, the total number of species per family ranged from 96 (Cornaceae) to 14,403 (Asteraceae) in the asterid clade and from 65 (Ulmaceae) to 12,978 (Fabaceae) in the rosid clade. Of all species in these families listed in the NCBI taxonomy, 5.35% had a *RefSeq* cpDNA sequence. For a comparison to the status of the same clade data from late 2021, see [28] (2022) for a similar presentation.

### 2.2. Distribution Assessment

We collected a summary of 5545 chloroplast sequences. Families with 20 or more sequences and genera with 10 or more sequences were used to calculate the distribution. The number of sequences used to calculate the length distribution and the statistics of the resulting boxplot values are shown in Table 1. The input and all result data are listed in Supplementary Table S1. The evaluation of the distributions was carried out for 54 families, which corresponds to 34.4% of all the families collected. These families contained 5076 sequences, which accounted for 91.5% of the total dataset. An assessment was performed for 108 genera, which contained 2695 sequences, representing 48.3% of the dataset. The number of sequences in the family records ranged from 21 to 543 sequences, and in the genera, it ranged from 10 to 178 sequences.

**Figure 2:**
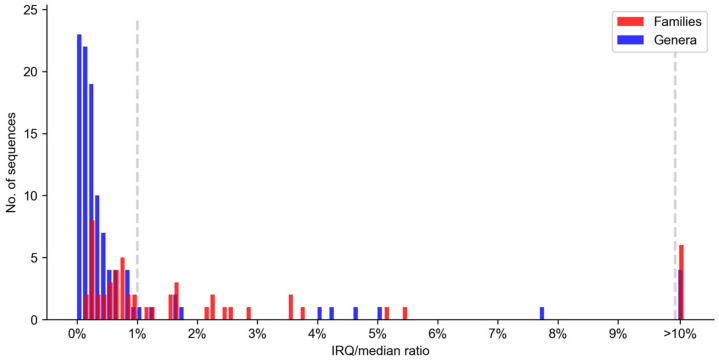
Histograms of IQR/median ratio values at the family (red) and genus levels (blue). Dashed vertical lines represent the thresholds used for family (10%) and genus (1%) levels.

The histograms of the resulting IQR/median values for the records of genera and families are shown in Figure 2. In the case of families, 30 records (55.56%) have an IQR/median value of less than 1%; this proportion is less than 5.5% in all (88.89%) except six families (11.11%). The distributions of the genera are generally narrower. As seen in the histogram, 64 of the genus records (59.26%) have an IQR/median of less than 0.3%, and 94 of the records (87.04%) have a ratio value of less than 1%. The rest of the genus groups (12.96%) have ratios greater than 1%. The results show that the distributions become narrower and have fewer outliers when lower taxonomic rank (i.e., genus) is used, which is to be expected.

After examining the IQR/median ratio values obtained, we decided to use 1% for genera and 10% for families as a threshold to consider a distribution as wide. Wide distributions were detected in 6 cases (11.11%) for families and in 14 cases (12.96%) for genera. The total number of outliers detected in the family distributions was 317, representing 6.25% of the species included in the analysis, with 40 families (74.07%) containing outliers. At the genus level, 69 genera (63.89%) contained outliers, and there was a total of 200 outliers, corresponding to 7.42% of the sequences analyzed.

According to the available sequences and the parameters studied (percentage of sequences with IQR/median > 1% and >10%, percentage of family/genus distributions with outliers, and percentage of sequences that were outliers at the family/genus level), both asterid and rosid clades provided fairly similar results (Table 1).

### 2.3. Examination of Wide Distributions

#### 2.3.1. Family Level

**Figure 3:**
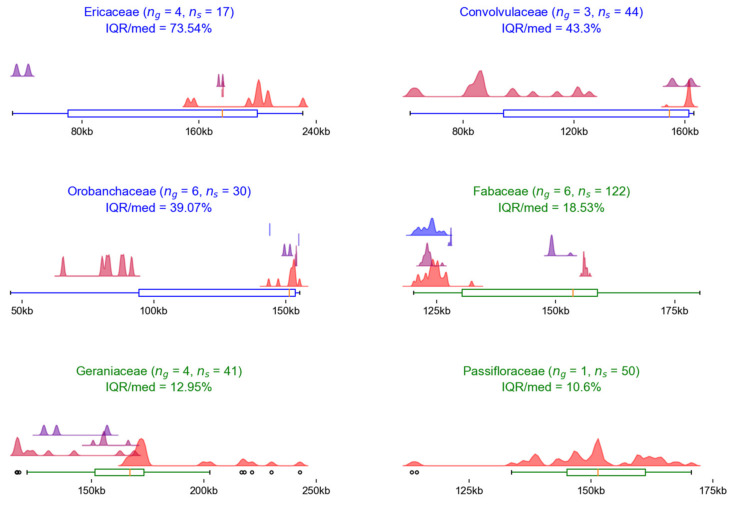
Box plots of the length distributions of the chloroplast genomes in the six families with the widest distributions. The length distributions of the largest genera within the families are represented by violin plots in different colors. A maximum of six of the largest genera with a number of sequences greater than 1 are shown. The numbers in brackets show the number of genera in the family (n_g_) and the number of sequences in the genera presented (n_s_). The title shows the IQR/median ratio for the family. The title and color of the boxplot represent the family clade: asterids (blue) or rosids (green). The numbers are arranged in descending order of IQR/median ratio values.

Table 2 lists families with distributions considered wide, showing an IQR/median ratio greater than 10%. The table contains the basic data; further details are shown in Figure 3, where the distribution is shown with a box plot and decomposed into the largest six genera, whose distributions are shown with violin plots. The results used to create the table and figure can be found in Table S1, worksheet “*WideFamilies*”.

Three of the six families with the widest chloroplast genome length distributions were those for which loss of inverted repeats (IR) was reported in some taxa: Ericaceae [29,30], Fabaceae [31,32], and Geraniaceae [15,33].

The occurrence of parasitic taxa was reported in two families with wide distributions. The Convolvulaceae family contains a single parasitic genus, *Cuscuta* [34,35], which has much shorter sequences (60–125 kbp) than those of the other Convolvulaceae genera (153–162 kbp). The Orobanchaceae family is considered the largest predominantly parasitic angiosperm family [36]. The results show that the sequence lengths of the Orobanchaceae family vary among genera (e.g., *Phelipanche* 62–63 kbp, *Orobanche* 65–91 kbp, *Cistanche* 94–102 kbp, *Aphyllon* 107–121 kbp, *Pedicularis* 143–155 kbp, *Rehmannia* 153–154 kbp), which is because this family contains both parasitic and hemiparasitic species.

The dataset of the family Passifloraceae contained mainly species of the genus *Passiflora*, in which extensive genomic alterations were detected, including inversions, gene and intron losses, and several independent IR expansions and contractions [37,38].

#### 2.3.2. Genus Level

The distributions of genera with IQR/median ratios greater than 1% are shown in Figure 3. The distribution is shown with a combined box and violin plot. The results used to create the figure can be found in Table S1, “*WideGenera*” worksheet.

For all genera showing a wide distribution of sequence lengths, some specific reasons for the variation in chloroplast genome length have been previously reported (Table 3). These reasons were the following: parasitic life form (e.g., *Cuscuta*), IR loss (e.g., *Erodium*, *Lathyrus*, *Medicago*, *Rhododendron*), IR expansions and contractions (e.g., *Passiflora*, *Pelargonium*), and polyphyly (e.g., *Amphilophium*, *Gentiana*, *Euphorbia*, *Lobelia*, *Peucedanum*, *Primula*, *Seseli*). The parasitic genus *Cuscuta* had the widest distribution of chloroplast genome length, ranging from 60 to 125 kbp. The results showed that three *Cuscuta* species (*C. exaltata*, *C. japonica*, and *C. reflexa*) with the longest sequences were hemiparasitic and belonged to the same paraphyletic group [35]. For the remaining genera, it is noteworthy that the IR changes seem to affect the distribution of sequence lengths more than the reported polyphyly of the genera in question. In addition, it is expected that the resolution of generic polyphyly will lead to the creation of new genera whose distribution will be much narrower.

**Figure 4:**
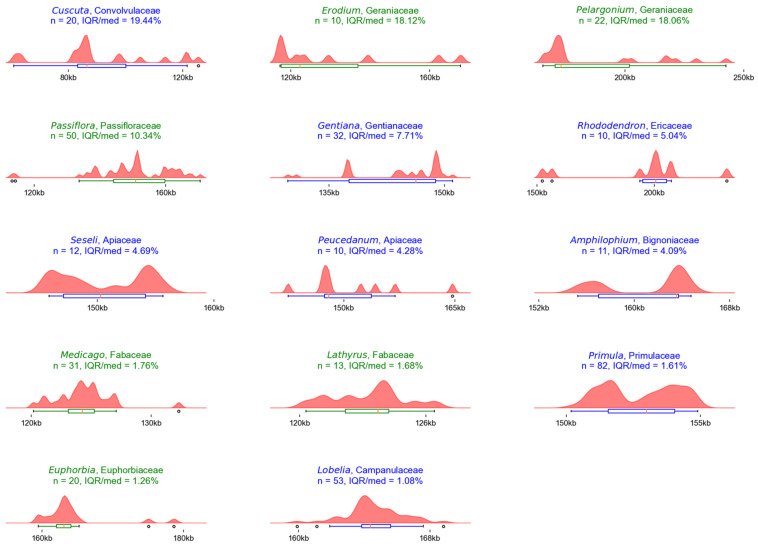
Composite box and violin plots of the length distributions of chloroplast genomes within genera showing wide distributions. The titles of the plots show the name of the genus and family, as well as the values for the number of sequences in the genus (n) and the IQR/median ratio of the distribution. The title and color of the boxplot represent the family clade: asterids (blue) or rosids (green). The genera are arranged in descending order of IQR/median ratio.

Apart from the fact that some genera showed a wide distribution, which can be explained by the biological phenomena mentioned above, some possible outliers were also detected, which can be clearly observed in Figure 4.

### 2.4. Outlier Detection at the Genus Level

Outliers were deetcted in 69 genera, representing 63.89% of the records where the length distributions were evaluated. Two hundred outliers were identified, which corresponded to 7.42% of the sequences used for the evaluation. For these species, we downloaded a summary of all available chloroplast sequences from the *GenBank* database. A total of 613 *GenBank* sequence summaries were acquired.

Note that the *GenBank* database contains both *RefSeq* sequences and submissions of the same sequences before they were included in the *RefSeq* database. Thus, 400 of the 613 sequences that we originally collected in the *RefSeq* database were identical sequences. These 213 alternative sequences belonged to 80 different species. Among them, we detected chloroplast sequences of 57 species (71.25%) whose length was closer to the median of the genus distribution than the original *RefSeq* sequence. A selection of cases where alternative *GenBank* sequences fit the genus distribution better than the original *RefSeq* sequence is provided in Table 4.

In genera with wide distributions, outliers were detected in seven datasets (Figure 4), with a total of 13 outlier sequences. Alternative sequences were detected for two species, both closer to the median of the genus distribution than the original sequences. Details of the results can be found in Table S1, worksheets “*Outliers*” and “*Alternatives*”.

## 3. Discussion

Our survey was prompted by the consideration that closely related species are more likely to share similar genome sizes and characteristics [16,51] and that a large amount of available cpDNA sequence data can be used to improve bioinformatic solutions for chloroplast assembly and annotation. In developing a method to quantify the expected proximity of related cpDNA sequences, the method must take into account potential differences arising from the technology used for sequence assembly in addition to the biological differences we wish to measure.

Given the large amount of data available, dissimilarities due to technology could be detected. These dissimilarities can range from problems of uniformity [52,53] to possible errors of assembly. Standardization problems, when discovered, can be resolved relatively easily by reformatting and reannotating the sequence data. On the other hand, sequence assembly inaccuracies are difficult to detect and virtually impossible to demonstrate without creating a new sequence assembly based on newer and presumably more accurate technologies and tools.

The problem of using existing data and the difficulty of detecting inadequacies in sequencing are related. To solve them, we need a quantification or model for the relationship between taxonomic proximity and differences in cpDNA sequences or some genomic characteristics. By default, genomic differences were detected by the alignment of sequences. In the case of cpDNA, this is not straightforward because the genome is circular and usually contains structures induced by inverted repeats (IRs). Therefore, the sequence format should be standardized [53]. However, a reliable IR identification method is still not available [28], while a different standardization approach would be required for the IR loss clades, probably based on conserved gene loci.

The study of the relationship between taxonomic proximity and differences in cpDNA sequence lengths was performed at two taxonomic levels for families and genera. Generally, results at the lower taxonomic level of genus are used by bioinformatics tools. The families studied mostly had narrow length distributions. The most extreme distributions were detected in parasitic families or those with IR losses or changes. Length distributions in the genera were generally very narrow, with more than half of the cases having an IQR/median ratio of less than 0.25%. This means that more than half of the genus sequences had lengths less than 200 base pairs from the median of the genus distribution. Only 12.96% of the genera had wide distributions as defined (IQR/median ratio > 1%). For all these wide distributions, there are some clear biological explanations, which are the main factors for the length variations: parasitic life form IR loss, IR expansions and contractions, and polyphyly.

Distribution outliers were also detected and tested with data from the *GenBank* database. In most cases (71.25%), the alternative sequences of the same species matched the expected distributions of the genera better than the original outlier sequences. This suggests that outlier detection is a promising method to identify suspect assemblies that require closer examination and possible correction.

We presented a strategy for using large amounts of existing cpDNA sequence data to derive a useful quantification of the relationship between species closeness and sequence length. The sequence length is well conserved at the genus level. Specifically, for chloroplast genomes, the property is also conserved at higher taxonomic levels, except in cases of clades with known high genome loss (IRs loss or alterations, parasitic clades). These results are useful for the development of specialized chloroplast bioinformatics tools. In addition, we have demonstrated a simple and promising method for identifying imperfect assemblies by combining outlier detection with checking data against sequences from other databases.

## 4. Materials and Methods

### 4.1. Data Acquisition

Summaries of chloroplast genome sequences were downloaded from the NCBI *RefSeq* database (https://www.ncbi.nlm.nih.gov/genome/browse#!/organelles/ (accessed on 22 October 2022)). We acquired summaries of all sequences available at that time for asterid and rosid clades. These clades were chosen as examples because they contain a sufficient number of sequences to demonstrate the utility of the bioinformatics pipeline we present. The sequence summaries included information on species name, accession number, genome length, and publication date. A total of 5545 sequence summaries were acquired. Additionally, for outlier species, summaries of alternative complete chloroplast sequences were downloaded from the NCBI *GenBank* database (https://www.ncbi.nlm.nih.gov/nucleotide (accessed on 22 October 2022)). Taxonomic data were retrieved from the NCBI Taxonomy Database (https://www.ncbi.nlm.nih.gov/taxonomy (accessed on 22 October 2022)).

### 4.2. Bioinformatics Pipeline

The research was based on the characteristics of the complete chloroplast sequence length distribution within a family or a genus. We used the descriptive statistics of box plots to represent the distributions. A box plot is a method of describing a dataset using five values: the median of the sample, the first and third quartiles, and the lower and upper whiskers. The whiskers are 1.5× interquartile ranges (IQRs), and the IQR is the distance between the upper and lower quartiles. Values that fell outside the whiskers were considered outliers [54]. As the main descriptor for the width of the distribution, we used the ratio of IQR and median values (IQR/median), which were presented as percentages.

We implemented a bioinformatics pipeline to acquire the data we need and store all the research results. The tool is controlled by arguments that determine the scope of data to be analyzed and the thresholds used in the calculations. The results of an analysis are saved in an Excel spreadsheet, and graphs based on the results obtained can be generated.

The analysis is carried out in four steps: data acquisition, distribution assessment, examination of the wide distributions, and outlier detection. Data acquisition was performed by querying the *RefSeq* database for summaries of the complete chloroplast sequences of selected species. The downloaded summary data are stored in the Excel worksheet “*RefSeq*”. For this research, we used sequence data from the asterid and rosid clades.

The assessment of the length distributions of chloroplast genomes is performed within families and genera containing at least a certain number of sequences. The calculated distribution characteristics are stored in the Excel worksheet “*Distributions*”. In this research, we carried out an assessment for families with at least 20 sequences and genera with at least 10 sequences.

When examining taxa that have a wide distribution of chloroplast genome lengths, families and genera whose IQR/median value is above the specified threshold are filtered out. The filtered data are stored in the Excel worksheets “*WideAll*”, “*WideFamilies*”, and “*WideGenera*”. For families, the assessment data also include the distribution characteristics of their genera to check their influence on the family distribution. In our research, we chose a threshold of 1% for the distributions of genera and 10% for the distributions of families based on the obtained IQR/median values.

The outlier test detects sequences that are outliers in the distribution of their genera. For species that are considered outliers, the tool downloads a summary of all complete chloroplast sequences from the *GenBank* database. It then tests whether there is a sequence between those whose length is closer to the median of the genus distribution than that of the *RefSeq* sequence. The list of all detected outliers from the genus distribution is stored in the Excel worksheet “*Outliers*”, and the list of outliers where an alternative sequence is detected that is closer to the median of the distribution is saved in the worksheet “*Alternatives*”. Note that the *RefSeq* database is part of the *GenBank* database, and the *GenBank* database also contains versions of *RefSeq* sequences before they were included in *RefSeq*, so the query retrieves a summary for at least two sequences.

The pipeline to perform the analysis was implemented in Python using the Biopython package [55] to retrieve sequence summaries. Taxonomy analysis was performed using the ETE 3 [56] Python library. Figures were generated using the Python library Matplotlib [57]. The code is maintained in a public repository (https://github.com/CroP-BioDiv/cpdna_survey; accessed on 22 October 2022). The script receives arguments for the taxa to be analyzed, the minimum number of sequences to calculate the distribution, and the thresholds for the IQR/median ratio.

## Figures and Tables

**Table 1:** Total number of taxa and sequences collected and analyzed. Summary of the results on the distribution range and outliers.

Parameter	Family Level			Genus Level		
	Asterids	Rosids	Total	Asterids	Rosids	Total
Total number of taxa	68	89	157	690	905	1595
No. of taxa analyzed	26	28	54	49	59	108
No. sequences in analyses	2337	2739	5076	1285	1410	2695
Minimum no. of sequences	21	21	21	10	10	10
	Balsaminaceae	Ulmaceae		*Rhododendron*	*Glycine*	
Maximum no. of sequences	543	480	543	178	78	178
	Asteraceae	Fabaceae		*Solanum*	*Acer*	
Examination of wide distributions						
No. and % of sequences IQR/median > 1%	11	13	24	8	6	14
	(42.31%)	(46.43%)	(44.44%)	(16.33%)	(10.17%)	(12.96%)
No. and % of sequences IQR/median > 10%	3	3	6	1	3	4
	(11.54%)	(10.71%)	(11.11%)	(2.04%)	(5.08%)	(3.70%)
Outlier detection						
No. of distributions with outliers	18	22	40	31	38	69
	(69.23%)	(78.57%)	(74.07%)	(63.27%)	(64.41%)	(63.89%)
Total number of outliers	188	129	317	103	97	200
	(8.04%)	(4.71%)	(6.25%)	(8.02%)	(6.88%)	(7.42%)

**Table 2:** Families showing wide distributions of chloroplast genome lengths: number of genera with *RefSeq* sequences, number of sequences, IQR/median ratio, and list of genera with wide distributions.

Family	No. of Genera	No. of Sequences	IQR/Median Ratio	Genera Showing Wide Distributions
Ericaceae ^a^	9	22	73.54%	*Rhododendron*
Convolvulaceae ^a^	11	52	43.30%	*Cuscuta*
Orobanchaceae ^a^	22	49	39.07%	-
Fabaceae ^r^	223	480	18.53%	*Medicago*, *Lathyrus*
Geraniaceae ^r^	5	42	12.95%	*Pelargonium*, *Erodium*
Passifloraceae ^r^	4	53	10.60%	*Passiflora*

^a^ asterid clade. ^r^ rosid clade.

**Table 3:** Genera showing wide distributions of chloroplast genome lengths: family, number of sequences, IQR/median ratio, and reported factor responsible for variation in chloroplast genome length.

Genus	Family	No. of Sequences	IQR/Median Ratio	Reported Factor
*Cuscuta*	Convolvulaceae ^a^	20	19.44%	Parasitic life form [34,35]
*Erodium*	Geraniaceae ^r^	10	18.12%	IR loss [15,33]
*Pelargonium*	Geraniaceae ^r^	22	18.06%	IR expansions [15,39,40,41]
*Passiflora*	Passifloraceae ^r^	50	10.34%	IR expansions and contractions [37,38]
*Gentiana*	Gentianaceae ^a^	32	7.71%	Polyphyly [42]
*Rhododendron*	Ericaceae ^a^	10	5.04%	IR loss [29,30]
*Seseli*	Apiaceae ^a^	12	4.96%	Polyphyly [43]
*Peucedanum*	Apiaceae ^a^	10	4.28%	Polyphyly [43]
*Amphilophium*	Bignoniaceae ^a^	11	4.09%	Polyphyly [44]
*Medicago*	Fabaceae ^r^	31	1.76%	IR loss [31,32]
*Lathyrus*	Fabaceae ^r^	13	1.68%	IR loss [31,32]
*Primula*	Primulaceae ^a^	82	1.61%	Polyphyly [45,46,47]
*Euphorbia*	Euphorbiaceae ^r^	20	1.26%	Polyphyly [48,49]
*Lobelia*	Campanulaceae ^a^	53	1.08%	Polyphyly [50]

^a^ asterid clade. ^r^ rosid clade.

**Table 4:** Examples of *RefSeq* sequences where alternative *GenBank* sequences were detected that are closer to the median of the genus distribution. The accession number, sequence length, and publication date of the sequences are provided.

Species	Genus Median	*RefSeq* Sequence			Alternative Sequence		
		Accession	Length	Publication Date	Accession	Length	Publication Date
*Angelica sinensis*	146,962	NC_042826	142,485	25 June 2019	MW820164	146,952	5 September 2021
*Ficus auriculata*	160,363	NC_053837	162,558	26 March 2021	MZ662866	160,361	31 August 2022
*Fragaria mandshurica*	155,621	NC_018767	129,805	14 October 2012	MW537846	155,640	30 March 2022
*Fragaria vesca*	155,621	NC_018766	129,788	14 October 2012	KC507757	155,620	26 July 2016
*Vitis romanetii*	160,971	NC_056348	232,020	20 June 2021	MW592524	160,976	16 March 2022

## Data Availability

The data that support the findings of this study are openly available in the NCBI Nucleotide Database (https://www.ncbi.nlm.nih.gov/nucleotide (accessed on 22 October 2022)) and NCBI Taxonomy Database (https://www.ncbi.nlm.nih.gov/taxonomy (accessed on 22 October 2022)).

## Supplementary Materials

The following supporting information can be downloaded at: https://www.mdpi.com/article/10.3390/plants12020254/s1, Table S1: Excel file with worksheets for input data and all analyses results.
